# Generalized Marshall-Olkin exponentiated exponential distribution: Properties and applications

**DOI:** 10.1371/journal.pone.0280349

**Published:** 2023-01-18

**Authors:** Egemen Ozkan, Gulhayat Golbasi Simsek

**Affiliations:** Department of Statistics, Yildiz Technical University, Istanbul, Türkiye; Mustansiriyah University - College of Science, IRAQ

## Abstract

In this study, we propose a generalized Marshall-Olkin exponentiated exponential distribution as a submodel of the family of generalized Marshall-Olkin distribution. Some statistical properties of the proposed distribution are examined such as moments, the moment-generating function, incomplete moment, and Lorenz and Bonferroni curves. We give five estimators for the unknown parameters of the proposed distribution based on maximum likelihood, least squares, weighted least squares, and the Anderson-Darling and Cramer-von Mises methods of estimation. To investigate the finite sample properties of the estimators, a comprehensive Monte Carlo simulation study is conducted for the models with three sets of randomly selected parameter values. Finally, four different real data applications are presented to demonstrate the usefulness of the proposed distribution in real life.

## 1. Introduction

Statistical distributions are widely used to model data including survival analysis. Exponential, Weibull, Rayleigh, gamma, and lognormal distributions have central importance in the literature as they are among the most flexible distributions used in survival analysis. However, considering the unlimited number of data generation processes, these distributions alone may be insufficient for modeling. Thus, new distributions and distribution families have been derived in recent years by utilizing existing distributions with derivation methods including transformation, compounding, and exponentiation. The LBeta-G family [1], Kumaraswamy-G family [2], Topp-Leone G family [3], modified beta transmuted-G family [4], exponentiated Weibull distribution [5], Odd-Lidney half-logistic distribution [6], and extended Gumbel distribution [7] are examples of these types of derived distributions. Among others, the weighted exponential [8], Nadarajah-Haghighi exponential [9], exponentiated generalized linear exponential [10], exponentiated Weibull (EW) [11], exponentiated Weibull-Poisson (EWP) [12], extended exponential [13], α-power transformed generalized exponential [14], odd exponentiated half-logistic exponential [15], exponentiated additive Weibull [16], exponentiated Weibull-exponential [17], extended odd Weibull exponential (EOWEx) [18], and bimodal exponential [19] distributions are extensions of the exponential distribution frequently used in survival analysis. Exponentiated distributions are obtained by exponentiating existing distributions. Because they have more parameters, their model fits are better compared to baseline distributions. The idea of exponentiated distributions was first introduced by Lehmann [20]. Exponentiated gamma, exponentiated Weibull, exponentiated Gumbel, and exponentiated Frechet distributions are members of the class of distributions obtained by exponentiation [21]. One of the widely used exponentiated distributions is the exponentiated exponential (EE) distribution. It was introduced by Verhulst [22] following the definition of the general form by Ahuja and Nash [23] and subsequently named by Gupta et al. [24]. The EE distribution with *θ* and *β* parameters is shown by *EE*(*θ*, *β*). The probability density function (pdf) and cumulative distribution function (cdf) of this distribution are given as follows:

$$f(x)=\beta \theta \exp\left(-\theta x\right){\left[1-\exp\left(-\theta x\right)\right]}^{\beta -1}$$ (1)

and

$$F\left(x\right)={\left[1-\exp\left(-\theta x\right)\right]}^{\beta },$$ (2)

where *θ* > 0, *β* > 0, and *x* > 0. The EE distribution has a flexible structure in data modeling as it is able to have a decreasing or increasing hazard function depending on the shape parameter. The EE distribution is used in applications such as forecasting precipitation data, software reliability growth models for vital quality metrics, estimating the average life of power system equipment, and recovery rate modeling [25].

Marshall and Olkin [26] discovered a new way to add parameters to a distribution family and they proposed the Marshall-Olkin (MO) distribution family. Sankaran and Jayakumar [27] indicated that the MO family has an odds ratio function. Subsequently, Gillariose et al. [28] described the basic motivations of this distribution family as obtaining models that have more flexible skewness than symmetric distributions and acquiring heavy-tailed distributions relative to the baseline distributions. Finally, the most important motivation was said to be deriving more flexible models by providing various forms of hazard rate functions (HRFs) compared to the baseline distributions. Moreover, the MO family has an explicit interpretation with comprehensive ordering properties, including the pdf and HRF. There are many lifetime distributions in the literature obtained by means of the MO distribution family, such as the MO Frechet distribution [29], beta MO distribution family [30], MO generalized exponential distribution [31], MO alpha power distribution [32], and Weibull MO family [33]. Chesneau et al. [34] proposed a generalization of the MO family, which they called the generalized Marshall-Olkin (GMO) distribution. It is remarkable that their obtained model is more flexible than the original MO distribution family. The pdf and cdf of the GMO distribution family are as follows:

$$g\left(x\right)=\frac{\left(1-\alpha \right)\left(1-\lambda \right){\left[F\left(x\right)\right]}^{2}+2\alpha \left(1-\lambda \right)F\left(x\right)+\alpha \lambda }{{\left(\alpha +\left(1-\alpha \right)F\left(x\right)\right)}^{2}}f\left(x\right),\;x\in R,$$ (3)

$$G\left(x\right)=\frac{\lambda F\left(x\right)+\left(1-\lambda \right){\left[F\left(x\right)\right]}^{2}}{\left(\alpha +\left(1-\alpha \right)F\left(x\right)\right)},\;x\in R,$$ (4)

where *α*, *λ* ∈ (0,1], and *F*(*x*) and *f*(*x*) are the cdf and pdf functions of the baseline distribution, respectively. When *λ* = 1 is placed in Eq 4, the standard MO distribution family is obtained [26]. This study aims to propose a GMO exponentiated exponential distribution with EE baseline distribution, derived from the GMO family. We represent this GMO exponentiated exponential distribution as *GMO*–*EE* (*α*, *λ*, *θ*, *β*) with parameters *α*, *λ*, *θ*, and *β* hereafter. There are two main reasons for choosing the EE as the baseline distribution. First, the EE is more effective than the two-parameter Weibull and two-parameter gamma distributions in data analysis. Second, as mentioned before, the EE has both increasing and decreasing HRFs [25]. The first motivation for our new model arises from the easy acquisition of the reliability function and HRF, as the cdf is quite simple. The second motivation is that its decreasing, increasing, upside-down bathtub, bathtub-shaped, constant, and increasing-decreasing-increasing HRFs can be used effectively for data modeling, especially in reliability analysis, hydrological, biological, and engineering applications. The most important motivation for our model is that it can be used as an alternative to the Weibull, gamma, EE, and EWP models in the literature.

In this study, a submodel GMO-EE distribution is introduced, benefiting from the GMO distribution family. In Section 2, the survival function, HRF, quantile function, moment-generating function, and moments are obtained. Section 3 provides the maximum likelihood estimator (MLE), least squares estimator (LSE), weighted least squares estimator (WLSE), Cramer-von Mises estimator (CVME), and Anderson-Darling estimator (ADE) for unknown parameters of the GMO-EE distribution. In Section 4, a Monte Carlo simulation study is conducted to compare the performances of the estimators in terms of biases and mean square errors (MSEs) for each parameter. In Section 5, four real data applications are presented to show the applicability of the GMO-EE model in real life. The final section concludes the study.

## 2. GMO-EE distribution and its properties

**Fig 1:**
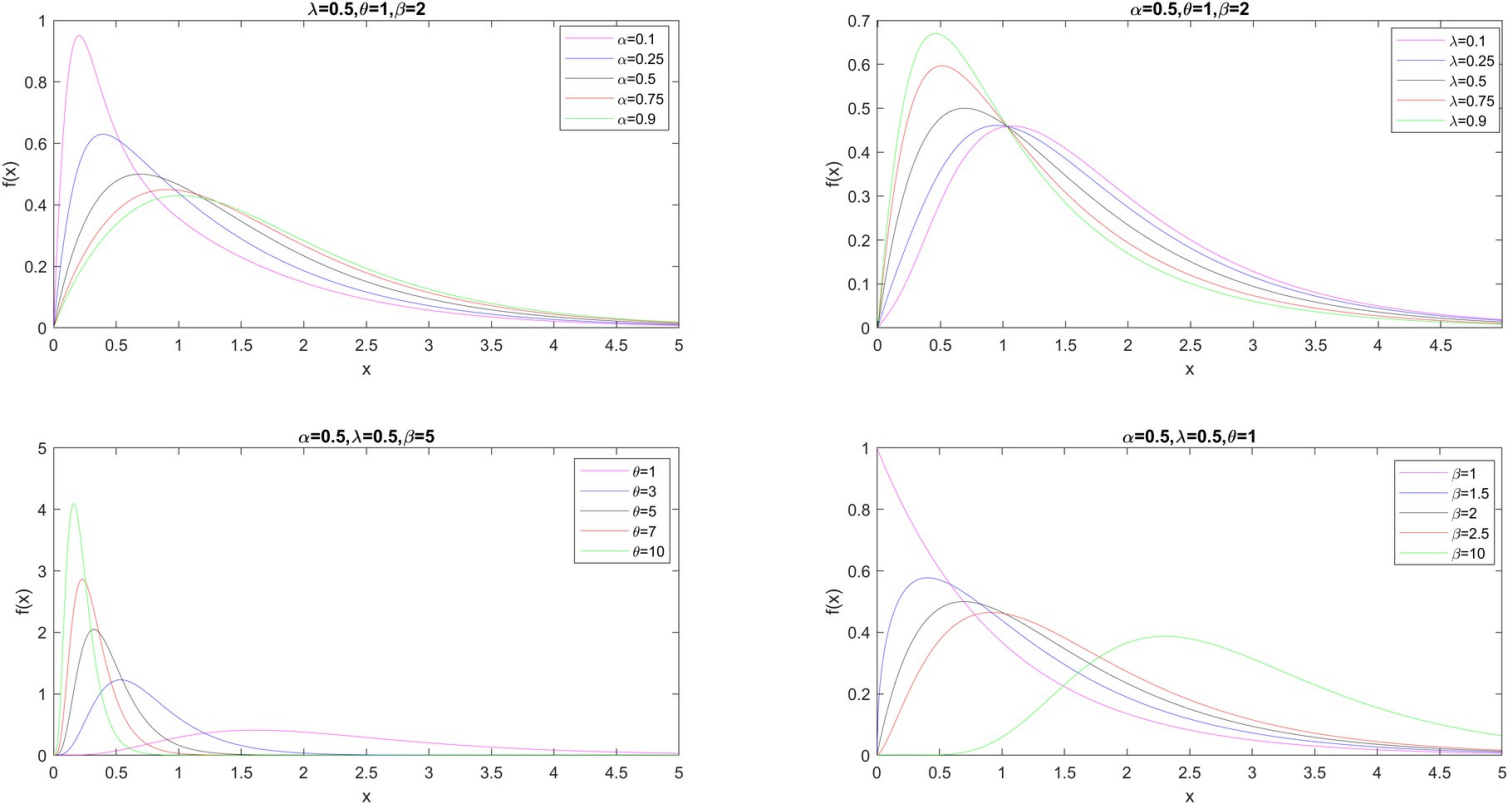
The pdf plots for selected parameter values.

In this section, we introduce a submodel GMO-EE survival distribution. Suppose that *X* is a random variable from the GMO-EE distribution. In this case, the pdf and cdf of *X* are given by the following:

$$\begin{array}{c} g\left(x,\eta \right)=\left(\frac{\left(1-\alpha \right)\left(1-\lambda \right){\left[1-\exp\left(-\theta x\right)\right]}^{2\beta }+2\alpha \left(1-\lambda \right){\left[1-\exp\left(-\theta x\right)\right]}^{\beta }+\alpha \lambda }{{\left[\alpha +\left(1-\alpha \right){\left(1-\exp\left(-\theta x\right)\right)}^{\beta }\right]}^{2}}\right)\times \\ \beta \theta \exp\left(-\theta x\right){\left[1-\exp\left(-\theta x\right)\right]}^{\beta -1} \end{array}$$ (5)

and

$$G\left(x,\eta \right)=\frac{\lambda {\left(1-\exp\left(-\theta x\right)\right)}^{\beta }+\left(1-\lambda \right){\left[1-\exp\left(-\theta x\right)\right]}^{2\beta }}{\alpha +\left(1-\alpha \right){\left(1-\exp\left(-\theta x\right)\right)}^{\beta }},$$ (6)

where **η** = (*α*, *λ*, *θ*, *β*) is the parameter vector and *α*, *λ* ∈ [0, 1), *θ*, *β* ∈ ℝ^+^ are the parameters. The GMO-EE distribution is reduced to the MO-EE distribution for *λ* = 1. The pdf plots for the various values of each parameter are shown in Fig 1 while the other parameters in the model are held constant.

The survival function and HRF for the GMO-EE distribution are respectively given as follows:

$$S\left(x,\eta \right)=\frac{\left[\alpha +\left(1-\lambda \right){\left(1-\exp\left(-\theta x\right)\right)}^{\beta }\right]\left[1-{\left(1-\exp\left(-\theta x\right)\right)}^{\beta }\right]}{\alpha +\left(1-\alpha \right){\left(1-\exp\left(-\theta x\right)\right)}^{\beta }}$$ (7)

and

$$h\left(x,\eta \right)=\frac{\left[\left(1-\alpha \right)\left(1-\lambda \right){\left(1-\exp\left(-\theta x\right)\right)}^{2\beta }+2\alpha \left(1-\lambda \right){\left(1-\exp\left(-\theta x\right)\right)}^{\beta }\right]\theta \beta \exp\left(-\theta x\right){\left(1-\exp\left(-\theta x\right)\right)}^{\beta -1}}{\left[\alpha +\left(1-\lambda \right){\left(1-\exp\left(-\theta x\right)\right)}^{\beta }\right]\left[\alpha +\left(1-\alpha \right){\left(1-\exp\left(-\theta x\right)\right)}^{\beta }\right]\left[1-{\left(1-\exp\left(-\theta x\right)\right)}^{\beta }\right]}.$$ (8)

**Fig 2:**
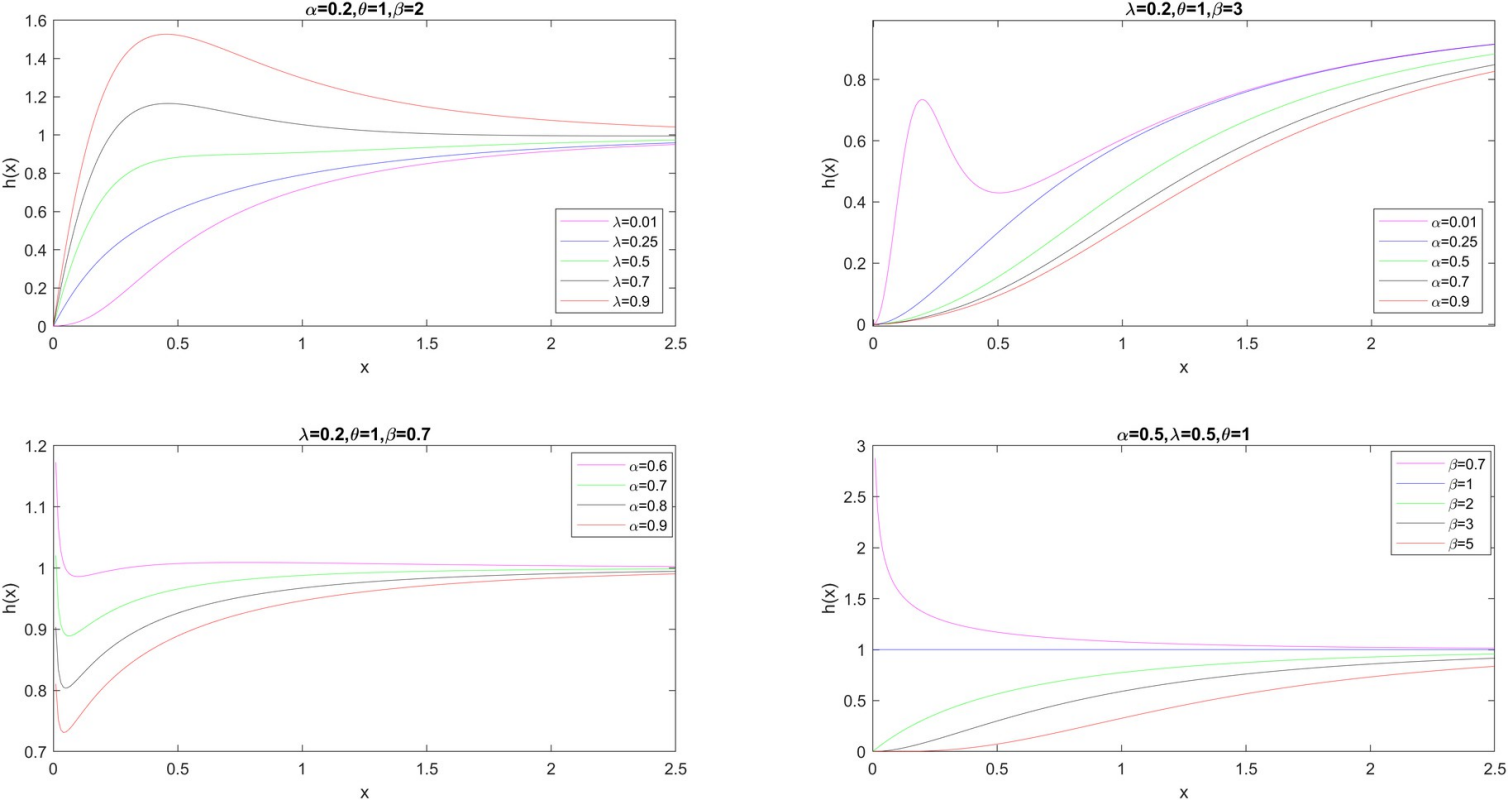
The HRF plots for selected parameter values.

Fig 2 shows the HRF plots for different values of parameters *α*, *λ*, *θ*, and *β* while the other parameters in the model are held constant.

### 2.1. Moment-generating function

In this subsection, the moment-generating function of the GMO-EE distribution is obtained. Let *X* be a random variable having distribution *GMO*–*EE*(*α*, *λ*, *θ*, *β*).

***Theorem 1***:

For any *x* and *α* provided that (1 – *α*)[1 –*F*(*x*)] ∈ (0,1), the pdf can be expanded to the series by using the following:

$$g\left(x\right)=f_{0}\left(x\right)+\sum_{l=0}^{\infty }\sum_{s=0}^{l+1}u_{l,s}f_{s}\left(x\right),$$ (9)

where *f*_*s*_(*x*) = (*s* + 1)*f*(*x*) (*F*(*x*))^*s*^ and $u_{l,s}=\left(\lambda -\alpha \right)\left(\begin{array}{c} l+1\\ s \end{array}\right){\left(1-\alpha \right)}^{l}{\left(-1\right)}^{s}$ [35].

The moment-generating function for the GMO-EE via Theorem 1 is given by the following:

$$M_{x}^{'}\left(t\right)=M_{x,0}^{*}\left(t\right)+\sum_{l=0}^{\infty }\sum_{s=0}^{l+1}u_{l,s}M_{x,s}^{*}\left(t\right),$$ (10)

where $M_{x,0}^{*}\left(t\right)=\int_{0}^{\infty }\exp\left(tx\right)f_{0}\left(x\right)dx$ and $M_{x,s}^{*}\left(t\right)=\int_{0}^{\infty }\exp\left(tx\right)f_{s}\left(x\right)dx$ are calculated as follows:

$$M_{x,0}^{*}\left(t\right)=E\left(e^{tx}\right)=\theta \beta \int_{0}^{\infty }\exp\left(t\left(x-\theta \right)\right){\left(1-\exp\left(-x\theta \right)\right)}^{\beta -1}dx,$$ (11)

$$M_{x,s}^{*}\left(t\right)=E\left(e^{tX}\right)=\left(s+1\right)\theta \beta \int_{0}^{\infty }\exp\left(t\left(x-\theta \right)\right){\left(1-\exp\left(-x\theta \right)\right)}^{\beta \left(s+1\right)-1}dx.$$ (12)

By using the transformation of *u* = exp(-*θx*) in Eqs 11 and 12, the following equations are respectively obtained:

$$M_{x,0}^{*}\left(t\right)=\beta \int_{0}^{1}u^{-\frac{t}{\theta }}{\left(1-u\right)}^{\beta -1}du=\frac{\Gamma \left(1-\frac{t}{\theta }\right)\Gamma \left(\beta +1\right)}{\Gamma \left(\beta +1-\frac{t}{\theta }\right)},$$ (13)

$$M_{x,s}^{*}\left(t\right)=\left(s+1\right)\beta \int_{0}^{1}u^{-\frac{t}{\theta }}{\left(1-u\right)}^{\beta \left(s+1\right)-1}du=\left(s+1\right)\frac{\Gamma \left(1-\frac{t}{\theta }\right)\Gamma \left(\beta \left(s+1\right)+1\right)}{\Gamma \left(\beta \left(s+1\right)+1-\frac{t}{\theta }\right)},$$ (14)

where $\Gamma \left(\alpha \right)=\int_{0}^{\infty }x^{\alpha -1}\exp\left(-x\right)dx$ is defined. Accordingly, the moment-generating function is expressed as

$$M_{x}^{'}\left(t\right)=\Gamma \left(1-\frac{t}{\theta }\right)\left(\frac{\Gamma \left(\beta +1\right)}{\Gamma \left(\beta +1-\frac{t}{\theta }\right)}+\sum_{l=0}^{\infty }\sum_{s=0}^{l+1}\left(s+1\right)\left(\lambda -\alpha \right)\left(\begin{array}{c} l+1\\ s \end{array}\right){\left(1-\alpha \right)}^{l}{\left(-1\right)}^{s}\frac{\Gamma \left(\beta \left(s+1\right)-1\right)}{\Gamma \left(\beta \left(s+1\right)+1-\frac{t}{\theta }\right)}\right).$$ (15)

### 2.2. Moments and incomplete moment

***Lemma 1***:

Binomial series expansion for any *n* > 0 is given by the following:

$${\left(a-x\right)}^{n}=\sum_{i=1}^{n}\left(\begin{array}{c} n\\ i \end{array}\right){\left(-1\right)}^{i}{\left(a\right)}^{n-i}{\left(x\right)}^{i},$$ (16)

where $\left(\begin{array}{c} n\\ i \end{array}\right)=\frac{n\left(n-1\right)\left(n-2\right)‥‥\left(n-i-1\right)}{i!},i=1,2,3,\ldots ,n$ [35].

Let us now consider the *r*^*th*^ moment of the GMO-EE distribution with parameters *α*, *λ*, *θ*, and *β*. The *r*^*th*^ moment is expressed as follows:

$$M_{r}^{'}=M_{r,0}^{*}+\sum_{l=0}^{\infty }\sum_{s=0}^{l+1}u_{l,r}M_{r,s}^{*},$$ (17)

where $M_{r,0}^{*}=\int_{0}^{\infty }x^{r}f_{0}\left(x\right)dx$ and $M_{r,s}^{*}=\int_{0}^{\infty }x^{r}f_{s}\left(x\right)dx$ are calculated as follows:

$$M_{r,0}^{*}=\theta \beta \int_{0}^{\infty }x^{r}\exp\left(-\theta x\right){\left(1-\exp\left(-\theta x\right)\right)}^{\beta -1}dx,$$ (18)

$$M_{r,s}^{*}=\left(s+1\right)\theta \beta \int_{0}^{\infty }x^{r}\exp\left(-\theta x\right){\left(1-\exp\left(-\theta x\right)\right)}^{\beta \left(s+1\right)-1}dx.$$ (19)

Using the series expansion in Eq 16, the integrals of Eqs 18 and 19 can be computed as follows:

$$\begin{array}{c} M_{r,0}^{*}=\theta \beta \int_{0}^{\infty }x^{r}\exp\left(-\theta x\right){\sum_{i=0}^{\beta -1}\left(\begin{array}{c} \beta -1\\ i \end{array}\right){\left(-1\right)}^{i}\left(\exp\left(-\theta x\right)\right)}^{i}\\ =\theta \beta \sum_{i=0}^{\beta -1}\left(\begin{array}{c} \beta -1\\ i \end{array}\right){\left(-1\right)}^{i}\int_{0}^{\infty }x^{r}\exp\left(-\theta x\left(i+1\right)\right)\\ =\beta \sum_{i=1}^{\beta -1}\left(\begin{array}{c} \beta -1\\ i \end{array}\right){\left(-1\right)}^{i}\frac{\Gamma \left(r+1\right)}{\theta^{r}{\left(i+1\right)}^{r+1}} \end{array}$$ (20)

and

$$\begin{array}{c} M_{r,s}^{*}=\left(s+1\right)\theta \beta \int_{0}^{\infty }x^{r}\exp\left(-\theta x\right)\sum_{i=1}^{\beta \left(s+1\right)-1}\left(\begin{array}{c} \beta \left(s+1\right)-1\\ i \end{array}\right){\left(-1\right)}^{i}{\left(\exp\left(-\theta x\right)\right)}^{i}dx\\ =\left(s+1\right)\theta \beta \sum_{i=1}^{\beta \left(s+1\right)-1}\left(\begin{array}{c} \beta \left(s+1\right)-1\\ i \end{array}\right){\left(-1\right)}^{i}\int_{0}^{\infty }x^{r}\exp\left(-\theta x\left(i+1\right)\right)dx\\ =\left(s+1\right)\beta \sum_{i=1}^{\beta \left(s+1\right)-1}\left(\begin{array}{c} \beta \left(s+1\right)-1\\ i \end{array}\right){\left(-1\right)}^{i}\frac{\Gamma \left(r+1\right)}{\theta^{r}{\left(i+1\right)}^{r+1}}. \end{array}$$ (21)

Thus, via Eqs 20 and 21, the *r*^*th*^ moment is obtained by:

$$\begin{array}{c} M_{r}^{'}=\frac{\beta \;\Gamma \left(r+1\right)}{\theta^{r}}\sum_{i=0}^{\beta -1}\left(\begin{array}{c} \beta -1\\ i \end{array}\right)\frac{{\left(-1\right)}^{i}}{{\left(i+1\right)}^{r+1}}+\frac{\beta \;\Gamma \left(r+1\right)}{\theta^{r}}\sum_{l=0}^{\infty }\sum_{s=0}^{l+1}\sum_{i=1}^{\beta \left(s+1\right)-1}\left(s+1\right)\times \\ \left(\lambda -\alpha \right)\left(\begin{array}{c} l+1\\ s \end{array}\right){\left(1-\alpha \right)}^{l}{\left(-1\right)}^{s+i}\left(\begin{array}{c} \beta \left(s+1\right)-1\\ i \end{array}\right)\frac{1}{{\left(i+1\right)}^{r+1}}. \end{array}$$ (22)

If *r* = 1 and *r* = 2 are taken in Eq 22, the first two moments are obtained in the form of $E\left(X\right)={M^{\prime }}_{1}$ and $E\left(X^{2}\right)={M^{\prime }}_{2}$. The variance is calculated as $Var\left(X\right)={M^{\prime }}_{2},-{\left({M^{\prime }}_{1}\right)}^{2}$through the first and second moment.

The *r*^*th*^ incomplete moment of random variable *X* having distribution *GMO*–*EE*(**η**) is given by the following:

$$\begin{array}{c} m_{r}\left(y\right)=\int_{0}^{y}x^{r}g\left(x\right)dx\\ =\frac{\beta }{\theta^{r}}\sum_{i=0}^{\beta -1}\left(\begin{array}{c} \beta -1\\ i \end{array}\right){\left(-1\right)}^{i}\frac{\Gamma \left(r+1,\theta y\left(i+1\right)\right)}{{\left(i+1\right)}^{r+1}}+\sum_{l=0}^{\infty }\sum_{s=0}^{l+1}\sum_{i=0}^{\beta \left(s+1\right)-1}\left(\lambda -\alpha \right)\left(\begin{array}{c} l+1\\ s \end{array}\right){\left(1-\alpha \right)}^{l}\times \\ \frac{\beta }{\theta^{r}}\left(s+1\right)\left(\begin{array}{c} \beta \left(s+1\right)-1\\ i \end{array}\right){\left(-1\right)}^{i+s}\frac{\Gamma \left(r+1,y\theta \left(i+1\right)\right)}{{\left(i+1\right)}^{r+1}}, \end{array}$$ (23)

where Γ(*a*, *b*) is an incomplete gamma function defined as $\Gamma \left(a,b\right)=\int_{0}^{x}t^{s-1}\exp\left(-t\right)dt.$

### 2.3. Bonferroni and Lorenz curves

The Bonferroni and Lorenz curves are basic methods used to analyze data in the areas of economics and reliability. The Bonferroni and Lorenz curves for the *GMO*–*EE*(**η**) distribution are respectively given by the following:

$$\begin{array}{c} B\left(p\right)=\frac{1}{p\mu }\int_{0}^{q}xf\left(x\right)dx\\ =\frac{\beta }{p\theta \mu }\sum_{i=1}^{\beta -1}\left(\begin{array}{c} \beta -1\\ i \end{array}\right){\left(-1\right)}^{i}\frac{\Gamma \left(2,q\theta \left(i+1\right)\right)}{{\left(i+1\right)}^{2}}+\frac{\beta }{p\theta \mu }\sum_{l=0}^{\infty }\sum_{s=0}^{l+1}\sum_{i=0}^{\beta \left(s+1\right)-1}\left(\lambda -\alpha \right)\left(\begin{array}{c} l+1\\ s \end{array}\right){\left(1-\alpha \right)}^{l}\times \\ \left(s+1\right)\left(\begin{array}{c} \beta \left(s+1\right)-1\\ i \end{array}\right){\left(-1\right)}^{s+i}\frac{\Gamma \left(2,\theta q\left(i+1\right)\right)}{{\left(i+1\right)}^{2}} \end{array}$$ (24)

and

$$\begin{array}{c} L\left(p\right)=\frac{1}{\mu }\int_{o}^{q}xg\left(x\right)dx\\ =\frac{\beta }{\theta \mu }\sum_{i=0}^{\beta -1}\left(\begin{array}{c} \beta -1\\ i \end{array}\right){\left(-1\right)}^{i}\frac{\Gamma \left(2,q\theta \left(i+1\right)\right)}{{\left(i+1\right)}^{2}}+\frac{\beta }{\theta \mu }\sum_{l=0}^{\infty }\sum_{s=0}^{l+1}\sum_{i=0}^{\beta \left(s+1\right)-1}\left(\lambda -\alpha \right)\left(\begin{array}{c} l+1\\ s \end{array}\right){\left(1-\alpha \right)}^{l}\times \\ \left(s+1\right)\left(\begin{array}{c} \beta \left(s+1\right)-1\\ i \end{array}\right){\left(-1\right)}^{s+i}\frac{\Gamma \left(2,\theta q\left(i+1\right)\right)}{{\left(i+1\right)}^{2}}, \end{array}$$ (25)

where *μ* is the first moment and *q* = *Q*(*p*) denotes the quantile function.

### 2.4. Quantile function

The quantile function for the distribution *GMO*–*EE*(*x*, **η**) = *p*, *p* ∈ (0,1) is obtained as follows:

$$x_{p}=-\frac{\log\left(-{\left(\frac{-\lambda +p-p\alpha +\sqrt{\lambda^{2}-2\lambda p\alpha +p^{2}-2p^{2}\alpha +p^{2}\alpha^{2}+4p\alpha }}{2\left(1-\lambda \right)}\right)}^{\frac{1}{\beta }}+1\right)}{\theta }.$$ (26)

## 3. Point estimation

### 3.1. Maximum likelihood estimation method

Let *X*_1_, *X*_2_,…, *X*_*n*_ be a random sample from *GMO*–*EE*(*x*, **η**). The log-likelihood function is given by the following:

$$\begin{array}{c} l\left(\eta \right)=\sum_{i=1}^{n}\log\left(\left(1-\alpha \right)\left(1-\lambda \right){\left(1-\exp\left(-\theta x_{i}\right)\right)}^{2\beta }+2\alpha \left(1-\lambda \right){\left(1-\exp\left(-\theta x_{i}\right)\right)}^{\beta }+\alpha \lambda \right)\\ +n\log\left(\theta \beta \right)-\theta \sum_{i=1}^{n}x_{i}+\left(\beta -1\right)\sum_{i=1}^{n}\log\left(1-\exp\left(-\theta x_{i}\right)\right)-2\sum_{i=1}^{n}\log\left(\alpha +\left(1-\alpha \right){\left(1-\exp\left(-\theta x_{i}\right)\right)}^{\beta }\right). \end{array}$$ (27)

Accordingly, the MLEs for the unknown parameters of **η** = (*α*, *λ*, *θ*, *β*) are obtained by solving the following equations:

$$\begin{array}{c} \frac{l\left(\eta \right)}{\partial \alpha }=\sum_{i=1}^{n}\frac{2\left(1-\lambda \right){\left(1-\exp\left(-\theta x_{i}\right)\right)}^{\beta }+\lambda -(1-\lambda ){\left(1-\exp\left(-\theta x_{i}\right)\right)}^{2\beta }}{\left(1-\alpha \right)\left(1-\lambda \right){\left(1-\exp\left(-\theta x_{i}\right)\right)}^{2\beta }+2\alpha \left(1-\lambda \right){\left(1-\exp\left(-\theta x_{i}\right)\right)}^{\beta }+\alpha \lambda }\\ -\sum_{i=1}^{n}\frac{\left(1-{\left(1-\exp\left(-\theta x_{i}\right)\right)}^{\beta }\right)}{\alpha +\left(1-\alpha \right){\left(1-\exp\left(-\theta x_{i}\right)\right)}^{\beta }}=0, \end{array}$$ (28)

$$\frac{l\left(\eta \right)}{\partial \lambda }=\sum_{i=1}^{n}\frac{\alpha -\left(1-\alpha \right){\left(1-\exp\left(-\theta x_{i}\right)\right)}^{2\beta }-2\alpha {\left(1-\exp\left(-\theta x_{i}\right)\right)}^{\beta }+\alpha }{\left(1-\alpha \right)\left(1-\lambda \right){\left(1-\exp\left(-\theta x_{i}\right)\right)}^{2\beta }+2\alpha \left(1-\lambda \right){\left(1-\exp\left(-\theta x\right)\right)}^{\beta }+\alpha \lambda }=0,$$ (29)

$$\begin{array}{l} \frac{l\left(\eta \right)}{\partial \theta }=\sum_{i=1}^{n}\frac{2\beta \left(1-\lambda \right)x_{i}\exp\left(-\theta x_{i}\right)\left[\left(1-\lambda \right){\left(1-\exp\left(-\theta x_{i}\right)\right)}^{2\beta }+\alpha {\left(1-\exp\left(-\theta x_{i}\right)\right)}^{\beta }\right]}{\left(1-\exp\left(-\theta x_{i}\right)\right)\left[\left(1-\alpha \right)\left(1-\lambda \right){\left(1-\exp\left(-\theta x_{i}\right)\right)}^{2\beta }+2\alpha \left(1-\lambda \right){\left(1-\exp\left(-\theta x_{i}\right)\right)}^{\beta }+\alpha \lambda \right]}\\ \;+\frac{n}{\theta }+\sum_{i=1}^{n}x_{i}+\sum_{i=1}^{n}\frac{\left(\beta -1\right)x\exp\left(-\theta x\right)}{1-\exp\left(-\theta x\right)}-\sum_{i=1}^{n}\frac{2\left(1-\alpha \right){\left(1-\exp\left(-\theta x\right)\right)}^{\beta }\beta x\exp\left(-\theta x\right)}{\left(1-\exp\left(-\theta x\right)\right)\left(\alpha +\left(1-\alpha \right){\left(1-\exp\left(-\theta x\right)\right)}^{\beta }\right)}=0, \end{array}$$ (30)

$$\begin{array}{l} \frac{l\left(\eta \right)}{\partial \beta }=\sum_{i=1}^{n}\frac{2\left(1-\lambda \right)\log\left(1-\exp\left(-\theta x_{i}\right)\right)\left[\left(1-\alpha \right){\left(1-\exp\left(-\theta x_{i}\right)\right)}^{2\beta }+\alpha {\left(1-\exp\left(-\theta x_{i}\right)\right)}^{\beta }\right]}{\left(1-\alpha \right)\left(1-\lambda \right){\left(1-\exp\left(-\theta x_{i}\right)\right)}^{2\beta }+2\alpha \left(1-\lambda \right){\left(1-\exp\left(-\theta x_{i}\right)\right)}^{\beta }+\alpha \lambda }\\ \;+\frac{n}{\beta }+\log\left(1-\exp\left(-\theta x_{i}\right)\right)-\sum_{i=1}^{n}\frac{2\left(1-\alpha \right){\left(1-\exp\left(-\theta x_{i}\right)\right)}^{\beta }\log\left(1-\exp\left(-\theta x_{i}\right)\right)}{\alpha +\left(1-\alpha \right){\left(1-\exp\left(-\theta x\right)\right)}^{\beta }}=0. \end{array}$$ (31)

### 3.2. Least square and weighted least square estimation

Let *X*_1_, *X*_2_, …, *X*_*n*_ be a random sample taken from *GMO*–*EE*(*x*, **η**). *X*_1:*n*_, *X*_2:*n*_, …, *X*_*n*:*n*_ are the order statistics in this sample. The expected value and variance of the empirical distribution function are as follows:

$$E\left[G\left(X_{i:n}\right)\right]=\frac{i}{n+1};\;i=1,2,\ldots ,n,$$ (32)

$$Var\left(G\left(X_{i:n}\right)\right)=\frac{i\left(n-i+1\right)}{{\left(n+1\right)}^{2}\left(n+2\right)};\;i=1,2,\ldots ,n.$$ (33)

Hence, the LSEs for the unknown parameters of distribution *GMO*–*EE*(*α*, *λ*, *θ*, *β*) are obtained by minimizing the objective function given as follows:

$$\Psi \left(\alpha ,\lambda ,\theta ,\beta \right)={\sum_{i=1}^{n}\left[G\left(x_{i:n},\alpha ,\lambda ,\theta ,\beta \right)-\frac{i}{n+1}\right]}^{2}.$$ (34)

Thus, the LSEs for the parameters, ${\hat{\alpha }}_{LSE},{\hat{\lambda }}_{LSE},{\hat{\theta }}_{LSE}$, and ${\hat{\beta }}_{LSE}$, are obtained by solving the systems of equations given by Eqs 35–38:

$$\frac{\partial \Psi \left(\alpha ,\lambda ,\theta ,\beta \right)}{\partial \alpha }=\sum_{i=1}^{n}{G^{\prime }}_{\alpha }\left(x_{i:n},\alpha ,\lambda ,\theta ,\beta \right)\left(G\left(x_{i:n},\alpha ,\lambda ,\theta ,\beta \right)-\frac{i}{n+1}\right)=0,$$ (35)

$$\frac{\partial \Psi \left(\alpha ,\lambda ,\theta ,\beta \right)}{\partial \lambda }=\sum_{i=1}^{n}{G^{\prime }}_{\lambda }\left(x_{i:n},\alpha ,\lambda ,\theta ,\beta \right)\left(G\left(x_{i:n},\alpha ,\lambda ,\theta ,\beta \right)-\frac{i}{n+1}\right)=0,$$ (36)

$$\frac{\partial \Psi \left(\alpha ,\lambda ,\theta ,\beta \right)}{\partial \theta }=\sum_{i=1}^{n}{G^{\prime }}_{\theta }\left(x_{i:n},\alpha ,\lambda ,\theta ,\beta \right)\left(G\left(x_{i:n},\alpha ,\lambda ,\theta ,\beta \right)-\frac{i}{n+1}\right)=0,$$ (37)

$$\frac{\partial \Psi \left(\alpha ,\lambda ,\theta ,\beta \right)}{\partial \beta }=\sum_{i=1}^{n}{G^{\prime }}_{\beta }\left(x_{i:n},\alpha ,\lambda ,\theta ,\beta \right)\left(G\left(x_{i:n},\alpha ,\lambda ,\theta ,\beta \right)-\frac{i}{n+1}\right)=0.$$ (38)

The WLSEs of parameters *α*, *λ*, *θ* and *β*, ${\hat{\alpha }}_{WLSE},{\hat{\lambda }}_{WLSE},{\hat{\theta }}_{WLSE}$, and ${\hat{\beta }}_{WLSE}$, are obtained by minimizing the objective function given by the following:

$$\omega \left(\alpha ,\lambda ,\theta ,\beta \right)={\sum_{i=1}^{n}w_{i}\left[G\left(X_{i:n}\right)-\frac{i}{n+1}\right]}^{2},$$ (39)

where *w*_*i*_ = (*n* + 1)^2^ (n + 1)/(*i*(*n*—*i* + 1)).

### 3.3. Anderson-Darling and Cramer-von Mises estimation

The Anderson-Darling method is based on the Anderson-Darling goodness-of-fit statistic proposed by Anderson and Darling [36]. Accordingly, ADEs for unknown parameters of the GMO-EE distribution are obtained by minimizing the following objective function:

$$A\left(\alpha ,\lambda ,\theta ,\beta \right)=-n-\frac{1}{n}\sum_{i=1}^{n}\left(2i-1\right)\left[\log\left(G\left(x_{i:n},\alpha ,\lambda ,\theta ,\beta \right)\right)+\log\left(1-G\left(x_{i:n}\alpha ,\lambda ,\theta ,\beta \right)\right)\right].$$ (40)

The Cramer-von Mises method, like the LSE and WLSE, is based on goodness-of-fit for the difference between the cdf and empirical distribution function. Thus, the Cramer-von Mises estimators ${\hat{\alpha }}_{CvME},{\hat{\lambda }}_{CvME},{\hat{\theta }}_{CvME}$, and ${\hat{\beta }}_{CvME}$ can be obtained by minimizing the following:

$$C\left(\alpha ,\lambda ,\theta ,\beta \right)=\frac{1}{12n}+{\sum_{i=1}^{n}\left[G\left(x_{i:n},\alpha ,\lambda ,\theta ,\beta \right)-\frac{2i-1}{2n}\right]}^{2}.$$ (41)

## 4. Simulation study

In this section, performances for the MLE, LSE, WLSE, ADE, and CvME estimators of the unknown parameters of the GMO-EE distribution are assessed according to mean biases and MSEs. The data are randomly generated from three GMO-EE models with selected parameter vectors **η**_**1**_ = (0.4,0.8,1,0.5), **η**_**2**_ = (0.5,0.7,3,0.9)0, and **η**_**3**_ = (0.2,0.5,2,0.75). The mean biases and MSEs are obtained in 5000 replications with sample sizes of 50, 100, 150, 200, 250, 500, 750, and 1000 for each of the models. However, the estimators of the parameters cannot be obtained in closed form. The BFGS, Nelder-Mead, CG, and L-BFGS-B [37] algorithms, which are numerical methods in R software [38], can be used to obtain the estimates of the parameters. The mean biases and MSEs in terms of the sample sizes and the five aforementioned estimators for the three models with **η**_1_, **η**_**2**_, and **η**_**3**_ are given in Tables 1–3, respectively.

**Table 1:** Mean bias and MSE for the model with *α* = 0.4, *λ* = 0.8, *θ* = 1, and *β* = 0.5.

		*Mean bias*				*MSE*			
*n*		$\hat{\alpha }$	$\hat{\lambda }$	$\hat{\theta }$	$\hat{\beta }$	$\hat{\alpha }$	$\hat{\lambda }$	$\hat{\theta }$	$\hat{\beta }$
50	*MLE*	-0.28068	-0.21289	0.12878	0.32917	0.09439	0.07028	0.13230	0.15473
	*LSE*	-0.26870	-0.14047	-0.04713	0.33943	0.15730	0.07187	0.15674	0.17698
	*WLSE*	-0.26969	-0.16348	-0.03111	0.29744	0.09006	0.05760	0.12407	0.13903
	*CvME*	-0.27618	-0.17265	0.01921	0.30419	0.08906	0.05734	0.11549	0.13320
	*ADE*	-0.30248	-0.17779	0.07342	0.40718	0.10553	0.05781	0.17959	0.23130
100	*MLE*	-0.23361	-0.18780	0.07709	0.20902	0.06971	0.05883	0.06699	0.06390
	*LSE*	-0.24670	-0.14643	-0.00722	0.25118	0.09513	0.06215	0.09679	0.09334
	*WLSE*	-0.23761	-0.15988	0.00178	0.21423	0.07112	0.05401	0.06952	0.06911
	*CvME*	-0.23717	-0.16180	0.01935	0.21035	0.06840	0.05206	0.06636	0.06327
	*ADE*	-0.26863	-0.17362	0.05957	0.28492	0.08600	0.05662	0.10442	0.11242
150	*MLE*	-0.19717	-0.14995	0.04835	0.15931	0.05473	0.04512	0.04279	0.03820
	*LSE*	-0.22552	-0.13246	-0.00074	0.20328	0.06985	0.05001	0.07593	0.06115
	*WLSE*	-0.21098	-0.13784	0.00098	0.16955	0.05798	0.04559	0.05071	0.04308
	*CvME*	-0.20837	-0.13612	0.01048	0.16651	0.05527	0.04267	0.04758	0.04003
	*ADE*	-0.24208	-0.15394	0.04736	0.22584	0.07274	0.05052	0.07987	0.07129
200	*MLE*	-0.18062	-0.11785	0.01368	0.13909	0.04630	0.03304	0.02856	0.02769
	*LSE*	-0.21446	-0.11487	-0.01862	0.17938	0.06220	0.04283	0.05886	0.04530
	*WLSE*	-0.19743	-0.11361	-0.01861	0.15163	0.05060	0.03504	0.03702	0.03232
	*CvME*	-0.19429	-0.11093	-0.01358	0.14822	0.04801	0.03305	0.03460	0.03009
	*ADE*	-0.22929	-0.13512	0.01914	0.19605	0.06402	0.04293	0.05951	0.05177
250	*MLE*	-0.17093	-0.10282	0.00943	0.13084	0.04047	0.02705	0.02294	0.02325
	*LSE*	-0.20438	-0.10650	-0.01344	0.16533	0.05411	0.03756	0.04949	0.03730
	*WLSE*	-0.18725	-0.10235	-0.01433	0.14197	0.04483	0.02930	0.02994	0.02687
	*CvME*	-0.18377	-0.09893	-0.01115	0.13926	0.04256	0.02746	0.02820	0.02536
	*ADE*	-0.21639	-0.12336	0.01746	0.17859	0.05693	0.03840	0.04993	0.04200
500	*MLE*	-0.13525	-0.08224	0.01692	0.10242	0.02754	0.02110	0.01410	0.01368
	*LSE*	-0.17353	-0.10339	0.01700	0.13296	0.04136	0.03339	0.02920	0.02328
	*WLSE*	-0.15569	-0.09385	0.01060	0.11435	0.03262	0.02460	0.01754	0.01672
	*CvME*	-0.15088	-0.08803	0.00957	0.11188	0.03081	0.02305	0.01690	0.01585
	*ADE*	-0.18185	-0.11441	0.03314	0.13973	0.04274	0.03379	0.03000	0.02516
750	*MLE*	-0.11531	-0.05141	-0.00069	0.08952	0.02175	0.01582	0.00892	0.01017
	*LSE*	-0.15256	-0.07956	0.00652	0.11372	0.03301	0.02581	0.01997	0.01660
	*WLSE*	-0.13710	-0.06916	-0.00002	0.10003	0.02611	0.01852	0.01152	0.01239
	*CvME*	-0.13262	-0.06358	-0.00158	0.09813	0.02471	0.01736	0.01107	0.01187
	*ADE*	-0.15855	-0.08770	0.01764	0.11813	0.03414	0.02614	0.02019	0.01764
1000	*MLE*	-0.09783	-0.02277	-0.01860	0.08534	0.01655	0.00958	0.00604	0.00883
	*LSE*	-0.13750	-0.05995	-0.00513	0.10715	0.02741	0.02043	0.01516	0.01440
	*WLSE*	-0.11941	-0.04202	-0.01577	0.09511	0.02066	0.01194	0.00841	0.01080
	*CvME*	-0.11557	-0.03711	-0.01717	0.09362	0.01959	0.01124	0.00818	0.01042
	*ADE*	-0.14216	-0.06537	0.00334	0.11094	0.02824	0.01933	0.01514	0.01507

**Table 2:** Mean bias and MSE for the model with *α* = 0.5, *λ* = 0.7, *θ* = 3, and *β* = 0.9.

		*Mean bias*				*MSE*			
*n*		$\hat{\alpha }$	$\hat{\lambda }$	$\hat{\theta }$	$\hat{\beta }$	$\hat{\alpha }$	$\hat{\lambda }$	$\hat{\theta }$	$\hat{\beta }$
50	*MLE*	-0.34566	-0.15146	0.00677	0.60609	0.14857	0.05449	0.48936	0.55477
	*LSE*	-0.36300	-0.07902	-0.41385	0.66166	0.16876	0.05246	0.81635	0.71067
	*WLSE*	-0.34991	-0.10611	-0.34523	0.55475	0.14735	0.05461	0.67605	0.52172
	*CvME*	-0.35018	-0.10466	-0.24528	0.56489	0.14420	0.04675	0.57542	0.47921
	*ADE*	-0.38821	-0.10309	-0.16052	0.81295	0.17133	0.04054	0.70152	0.95300
100	*MLE*	-0.28198	-0.16552	0.01055	0.35475	0.11357	0.06027	0.25390	0.21713
	*LSE*	-0.30520	-0.09688	-0.23223	0.45754	0.13920	0.05504	0.45554	0.34850
	*WLSE*	-0.29544	-0.13430	-0.16808	0.37014	0.11764	0.05688	0.33056	0.24228
	*CvME*	-0.29324	-0.13391	-0.13084	0.36300	0.11356	0.05411	0.30408	0.21771
	*ADE*	-0.33244	-0.12564	-0.09339	0.53200	0.14016	0.04755	0.41361	0.43109
150	*MLE*	-0.25939	-0.17702	0.00917	0.26147	0.09960	0.06560	0.17155	0.12454
	*LSE*	-0.27339	-0.11569	-0.16199	0.35557	0.13815	0.06708	0.30928	0.22372
	*WLSE*	-0.27616	-0.16345	-0.10978	0.28091	0.10530	0.06761	0.21463	0.15133
	*CvME*	-0.27159	-0.15454	-0.09228	0.27695	0.10064	0.06100	0.20430	0.13429
	*ADE*	-0.30060	-0.14182	-0.06659	0.40726	0.12446	0.05311	0.28670	0.26729
200	*MLE*	-0.23332	-0.17000	0.01844	0.21561	0.08888	0.06180	0.14380	0.09068
	*LSE*	-0.26228	-0.13427	-0.09816	0.31242	0.11443	0.06088	0.25047	0.17801
	*WLSE*	-0.25505	-0.16757	-0.05999	0.24203	0.09671	0.06423	0.17111	0.11598
	*CvME*	-0.25206	-0.16278	-0.05239	0.23454	0.09204	0.06153	0.16711	0.10277
	*ADE*	-0.28314	-0.15357	-0.02653	0.35121	0.11633	0.05625	0.23947	0.20618
250	*MLE*	-0.21808	-0.16684	0.01640	0.18197	0.08095	0.06031	0.11480	0.06843
	*LSE*	-0.24281	-0.13116	-0.08838	0.26619	0.11075	0.06333	0.20424	0.13449
	*WLSE*	-0.24005	-0.16742	-0.05158	0.20347	0.08805	0.06509	0.13894	0.08683
	*CvME*	-0.23587	-0.16003	-0.04570	0.19951	0.08367	0.06093	0.13661	0.07838
	*ADE*	-0.26466	-0.15044	-0.03014	0.29777	0.10647	0.05633	0.19496	0.15350
500	*MLE*	-0.16942	-0.12682	-0.01680	0.11043	0.05671	0.04660	0.06194	0.02733
	*LSE*	-0.21021	-0.11818	-0.07219	0.18232	0.08936	0.05816	0.11738	0.06389
	*WLSE*	-0.20490	-0.14241	-0.04973	0.13510	0.06498	0.05245	0.07519	0.03752
	*CvME*	-0.19878	-0.13533	-0.05032	0.13111	0.06162	0.05004	0.07483	0.03419
	*ADE*	-0.22698	-0.13412	-0.04145	0.19813	0.08290	0.05187	0.11233	0.07027
750	*MLE*	-0.14770	-0.10984	-0.01937	0.08707	0.04666	0.04131	0.04115	0.01616
	*LSE*	-0.20142	-0.11769	-0.05432	0.15270	0.07162	0.05238	0.08127	0.04145
	*WLSE*	-0.18389	-0.12805	-0.04020	0.10953	0.05453	0.04688	0.05070	0.02266
	*CvME*	-0.17827	-0.12248	-0.04130	0.10610	0.05171	0.04519	0.05080	0.02100
	*ADe*	-0.21514	-0.13144	-0.03313	0.16314	0.07093	0.05017	0.07837	0.04497
1000	*MLE*	-0.13367	-0.10333	-0.01148	0.07330	0.04124	0.03964	0.03309	0.01146
	*LSE*	-0.18694	-0.11393	-0.03964	0.13289	0.06514	0.05035	0.06399	0.03101
	*WLSE*	-0.17228	-0.12460	-0.02598	0.09604	0.04921	0.04407	0.04057	0.01645
	*CvME*	-0.16716	-0.11969	-0.02762	0.09281	0.04670	0.04286	0.04062	0.01522
	*ADE*	-0.19835	-0.12500	-0.02329	0.14128	0.06489	0.04897	0.06266	0.03342

**Table 3:** Mean bias and MSE for the model with α=0.2,λ=0.5,θ=2, and β=0.75.

		*Mean bias*				*MSE*			
*n*		$\hat{\alpha }$	$\hat{\lambda }$	$\hat{\theta }$	$\hat{\beta }$	$\hat{\alpha }$	$\hat{\lambda }$	$\hat{\theta }$	$\hat{\beta }$
50	*MLE*	-0.08729	0.01898	0.06158	0.36224	0.02545	0.02719	0.26641	0.22555
	*LSE*	-0.09775	0.07515	-0.18820	0.39060	0.02577	0.04029	0.40202	0.26906
	*WLSE*	-0.08453	0.05805	-0.16352	0.32183	0.02326	0.04197	0.32152	0.20409
	*CvME*	-0.08320	0.05372	-0.09287	0.32664	0.02219	0.03589	0.28176	0.18923
	*ADE*	-0.11314	0.05729	-0.01105	0.48436	0.02573	0.03096	0.39782	0.36162
100	*MLE*	-0.05482	0.00727	0.00484	0.19660	0.01878	0.02785	0.11746	0.07994
	*LSE*	-0.07591	0.04537	-0.11243	0.25280	0.01872	0.03788	0.22379	0.11937
	*WLSE*	-0.06138	0.02784	-0.09547	0.19628	0.01690	0.03660	0.15953	0.08326
	*CvME*	-0.05925	0.02783	-0.07337	0.19329	0.01579	0.03382	0.14257	0.07503
	*ADE*	-0.08993	0.03053	-0.01675	0.30082	0.01796	0.03083	0.21762	0.14846
150	*MLE*	-0.03255	0.01638	-0.01078	0.13770	0.01746	0.02615	0.08852	0.04673
	*LSE*	-0.05002	0.04803	-0.09206	0.18964	0.01894	0.03558	0.16853	0.07596
	*WLSE*	-0.03658	0.02644	-0.07694	0.13960	0.01807	0.03464	0.11689	0.05285
	*CvME*	-0.03600	0.02797	-0.06664	0.13653	0.01558	0.03286	0.10896	0.04735
	*ADE*	-0.06368	0.03457	-0.02606	0.22200	0.01731	0.02940	0.16288	0.09017
200	*MLE*	-0.01876	0.02062	-0.01745	0.09919	0.01621	0.02487	0.07138	0.02987
	*LSE*	-0.03499	0.04582	-0.07871	0.14420	0.01806	0.03398	0.13723	0.05132
	*WLSE*	-0.02926	0.02911	-0.06376	0.10774	0.01480	0.03118	0.09454	0.03308
	*CvME*	-0.02537	0.02579	-0.05854	0.10002	0.01422	0.03164	0.08905	0.03149
	*ADE*	-0.04763	0.03382	-0.02807	0.16861	0.01623	0.02885	0.13262	0.05949
250	*MLE*	-0.01282	0.04530	-0.05548	0.09095	0.01272	0.02384	0.05741	0.02368
	*LSE*	-0.02781	0.06725	-0.10518	0.13197	0.01536	0.03326	0.11498	0.04029
	*WLSE*	-0.01963	0.05475	-0.09250	0.09967	0.01224	0.02719	0.07711	0.02585
	*CvME*	-0.01780	0.05535	-0.08908	0.09583	0.01120	0.02712	0.07295	0.02402
	*ADE*	-0.03935	0.05507	-0.06304	0.15147	0.01342	0.02807	0.10843	0.04612
500	*MLE*	0.00548	0.03977	-0.04660	0.04903	0.01109	0.01865	0.03068	0.00990
	*LSE*	-0.00483	0.05370	-0.07235	0.07718	0.01422	0.02782	0.06090	0.01871
	*WLSE*	-0.00283	0.03980	-0.06184	0.05719	0.01037	0.01990	0.03834	0.01155
	*CvME*	0.00045	0.04337	-0.06272	0.05454	0.00998	0.02000	0.03778	0.01073
	*ADE*	-0.01283	0.04572	-0.05074	0.08724	0.01260	0.02468	0.05817	0.02042
750	*MLE*	0.01770	0.03929	-0.03921	0.02770	0.01046	0.01695	0.02225	0.00589
	*LSE*	0.01468	0.05417	-0.05972	0.04588	0.01489	0.02595	0.04175	0.01088
	*WLSE*	0.01253	0.03944	-0.05029	0.03267	0.01044	0.01873	0.02693	0.00698
	*CvME*	0.01544	0.04246	-0.05143	0.03065	0.01034	0.01905	0.02663	0.00664
	*ADE*	0.00809	0.04769	-0.04504	0.05266	0.01333	0.02354	0.04005	0.01158
1000	*MLE*	0.00945	0.01460	0.01799	0.00427	0.00945	0.01460	0.01799	0.00427
	*LSE*	0.01469	0.02370	0.03341	0.00768	0.01469	0.02370	0.03341	0.00768
	*WLSE*	0.00948	0.01559	0.02147	0.00480	0.00948	0.01559	0.02147	0.00480
	*CvME*	0.00978	0.01704	0.02150	0.00493	0.00978	0.01704	0.02150	0.00493
	*ADE*	0.01322	0.02169	0.03224	0.00800	0.01322	0.02169	0.03224	0.00800

When Tables 1–3 are examined, it is seen that the mean biases and MSEs decrease steadily as the number of samples increases. In addition, with the increase in sample size, the mean biases and MSEs approach zero, as expected. For these models, smaller values of mean biases and MSEs are mostly obtained with the MLE. When evaluated in general, the estimators give similar results.

## 5. Real data analysis

In this section, four real data applications are presented to compare the fits of the GMO-EE distribution and competing distributions. For this purpose, some comparative statistics such as the Cramer-von Mises (CvM), Kolmogorov-Smirnov (K-S), and Anderson-Darling (AD) test statistics with their p-values are applied for the four datasets together with Akaike’s information criterion (AIC) and -2 × log-likelihood values.

### 5.1. Dataset 1

**Fig 3:**
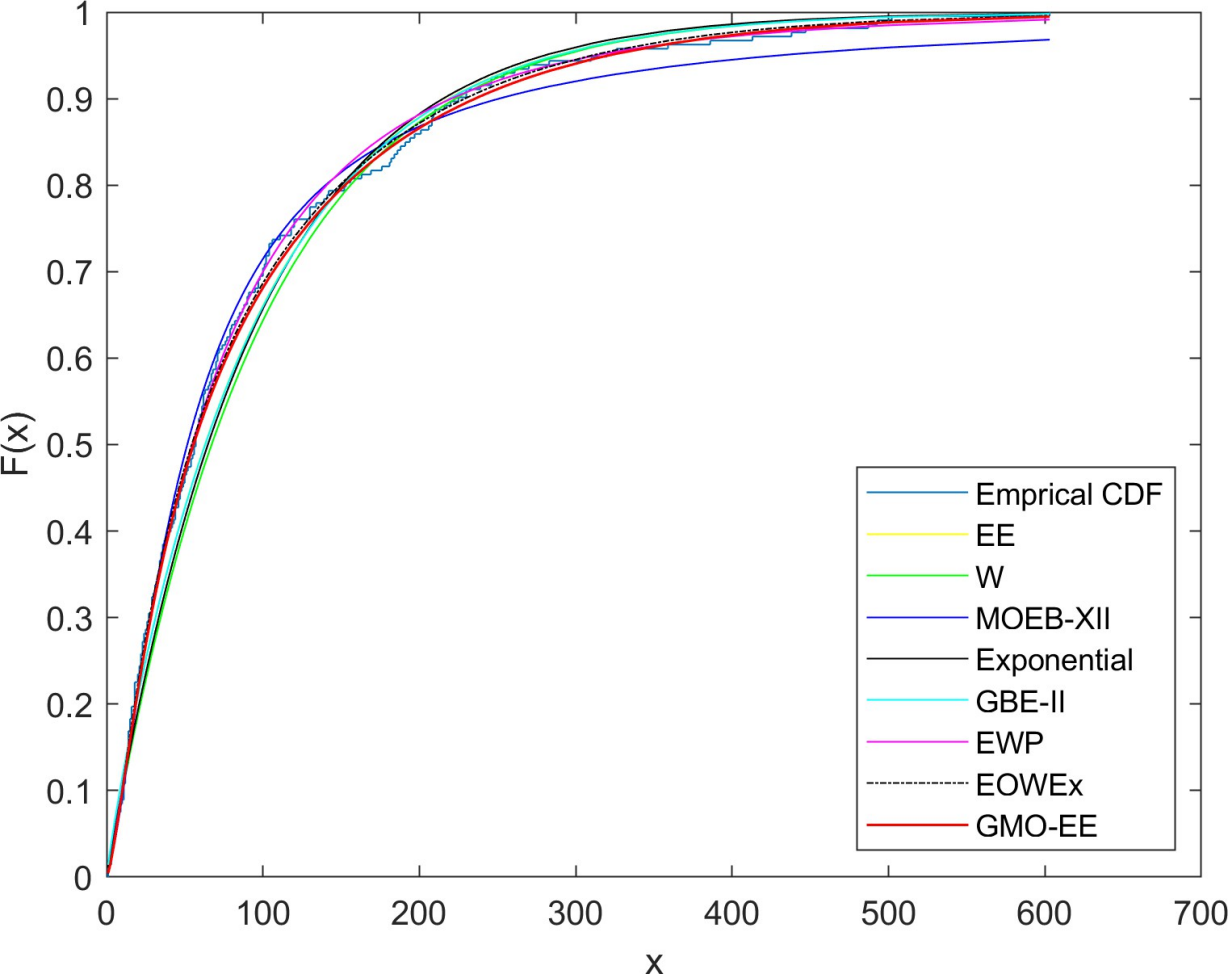
Fitted cdf plots for dataset 1.

The first dataset is attained from the number of successive failures of the air conditioning systems of the 13 members of a fleet of Boeing 720 jet airplanes. This dataset has been used in previous studies [39, 40]. The dataset consists of the following values: 194, 413, 90, 74, 55, 23, 97, 50, 359, 50, 130, 487, 102, 15, 14, 10, 57, 320, 261, 51, 44, 9, 254, 493, 18, 209, 41, 58, 60, 48, 56, 87, 11, 102, 12, 5, 100, 14, 29, 37, 186, 29, 104, 7, 4, 72, 270, 283, 7, 57, 33, 100, 61, 502, 220, 120, 141, 22, 603, 35, 98, 54, 181, 65, 49, 12, 239, 14, 18, 39, 3, 12, 5, 32, 9, 14, 70, 47, 62, 142, 3, 104, 85, 67, 169, 24, 21, 246, 47, 68, 15, 2, 91, 59, 447, 56, 29, 176, 225, 77, 197, 438, 43, 134, 184, 20, 386, 182, 71, 80, 188, 230, 152, 36, 79, 59, 33, 246, 1, 79, 3, 27, 201, 84, 27, 21, 16, 88, 130, 14, 118, 44, 15, 42, 106, 46, 230, 59, 153, 104, 20, 206, 5, 66, 34, 29, 26, 35, 5, 82, 5, 61, 31, 118, 326, 12, 54, 36, 34, 18, 25, 120, 31, 22, 18, 156, 11, 216, 139, 67, 310, 3, 46, 210, 57, 76, 14, 111, 97, 62, 26, 71, 39, 30, 7, 44, 11, 63, 23, 22, 23, 14, 18, 13, 34, 62, 11, 191, 14, 16, 18, 130, 90, 163, 208, 1, 24, 70, 16, 101, 52, 208, and 95. The dataset is fitted to GMO-EE, Weibull (W), EE [24], Marshall-Olkin Extended Burr Type XII (MOEBXII) [41], generalized binomial exponential-II (GBE-II) [42], EOWEx [18], EWP [12], and exponential distributions. MLEs with standard errors for unknown parameters of the fitted distributions and the comparative statistics are given in Tables 4 and 5, respectively. The cdf plots of the fitted distributions are shown in Fig 3.

**Table 4:** Parameter estimates (standard errors) for dataset 1.

	$\hat{\alpha }$	$\hat{\lambda }$	$\hat{\theta }$	$\hat{\beta }$
GMO-EE	0.1744 (0.1105)	0.7161(0.1834)	0.008(0.0014)	1.412(0.2170)
EWP	0.0076(1.4384)	0.0390(0.0543)	0.5561(0.1947)	2.8174(1.8256)
EOWEx	1.3954(0.2185)	0.0209(0.0052)	3.4321(1.3457)	-
MOEBXII	239.17(196.7109)	1.4003(4.0277)	0.9921(2.9693)	-
GBE-II	0.9267(0.0827)	0.0102(0.0017)	0.0056(0.2852)	-
EE	0.0100 (0.0009)	0.9275(0.0827)	-	-
W	0.9245 (0.0482)	89.5575(7.0175)	-	-
Exponential	0.0107(0.0008)	-	-	-

**Table 5:** Selection criteria statistics for dataset 1.

Distribution	-2log	AIC	AD	CvM	K-S	p(AD)	p(CvM)	p(K-S)
GMO-EE	**2347.32**	2355.32	**0.2030**	**0.0286**	**0.0307**	**0.9896**	**0.9805**	**0.9878**
EWP	2349.26	2357.26	0.2596	0.0345	0.0390	0.9650	0.9593	0.9009
EOWEx	2347.37	**2353.38**	0.2136	0.0308	0.0390	0.9862	0.9736	0.9602
MOEBXII	2361.12	2367.12	0.6385	0.0710	0.0420	0.6122	0.7459	0.8470
GBE-II	2356.81	2362.81	1.1881	0.2138	0.0641	0.2721	0.2423	0.3457
EE	2356.81	2360.81	1.1978	0.2165	0.0644	0.2684	0.2377	0.3396
W	2355.17	2359.17	0.8246	0.1275	0.0520	0.4635	0.4663	0.6113
Exponential	2357.53	2359.53	1.6919	0.1157	0.0726	0.1367	0.1157	0.2112

As seen from Table 5, the GMO-EE distribution outperforms the one-parameter exponential, two-parameter W and EE, and three-parameter MOEBXII and GBE-II distributions. Satisfactory and comparable model fits are provided by the three-parameter EOWEx and four-parameter EWP, while the best results are obtained by the GMO-EE except for the smaller AIC value of the EOWEx.

### 5.2. Dataset 2

**Fig 4:**
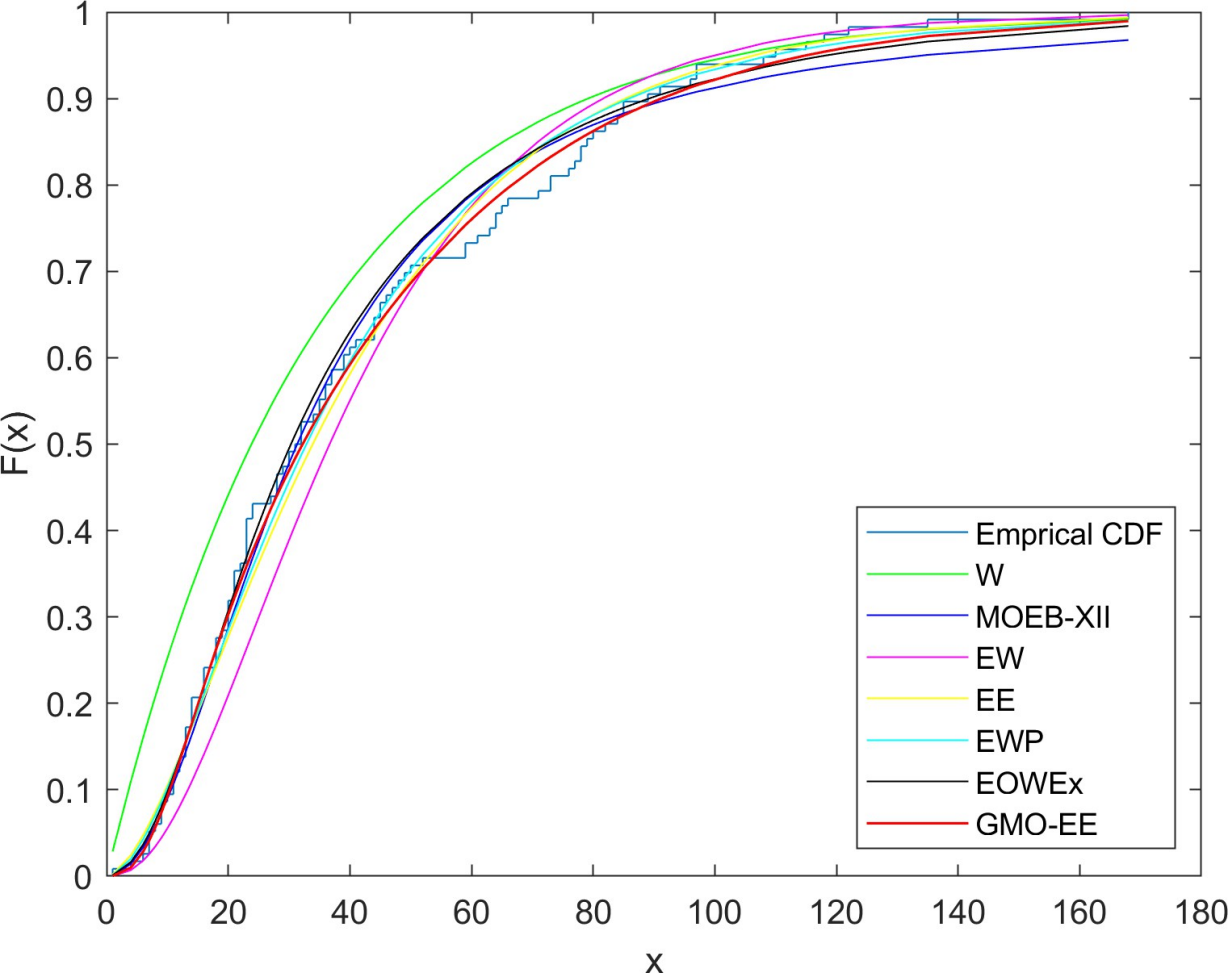
Fitted cdf plots for dataset 2.

The second dataset includes daily ozone level measurements in New York in May-September 1973. These data were used in previous studies [43, 44]. The dataset consists of the following values: 41, 36, 12, 18, 28, 23, 19, 8, 7, 16, 11, 14, 18, 14, 34, 6, 30, 11, 1, 11, 4, 32, 23, 45, 115, 37, 29, 71, 39, 23, 21, 37, 20, 12, 13, 135, 49, 32, 64, 40, 77, 97, 97, 85, 10, 27, 7, 48, 35, 61, 79, 63, 16, 80, 108, 20, 52, 82, 50, 64, 59, 39, 9, 16, 78, 35, 66, 122, 89, 110, 44, 28, 65, 22, 59, 23, 31, 44, 21, 9, 45, 168, 73, 76, 118, 84, 85, 96, 78, 73, 91, 47, 32, 20, 23, 21, 24, 44, 21, 28, 9, 13, 46, 18, 13, 24, 16, 13, 23, 36, 7, 14, 30, 14, 18, and 20. We analyze this dataset to compare the GMO-EE with the W, MOEBXII [41], EE [24], GBE-II [42], EOWEx [18], EWP [12], and EW [11] distributions. The results of this analysis are given in Tables 6 and 7, and the cdf plots of the fitted distributions are depicted in Fig 4. As seen from Table 7, the best fitted model is the GMO-EE according to all selection criteria except for the smaller AIC value of the EE.

**Table 6:** Parameter estimates (standard errors) for dataset 2.

	$\hat{\alpha }$	$\hat{\lambda }$	$\hat{\theta }$	$\hat{\beta }$
GMO-EE	0.1121(0.0958)	0.5427(0.1571)	0.030(0.0044)	2.8284(0.6450)
EWP	70.6413(159.4820)	0.0516(0.0324)	0.8054(0.2253)	0.0404(0.0982)
EOWEx	1.9237(0.4617)	0.0283(0.0063)	2.4251(1.2859)	-
MOEBXII	1062.58(606.767)	34.4062(38.9027)	0.0588(0.0665)	-
GBE-II	1.7962(0,2460)	0.0338(0,0197)	0.0082(1,1555)	-
EW	0.8350(0.2429)	21.1821(13.1199)	2.5804(1.6124)	-
EE	0.0336(0.0035)	1.7960(0.2455)	-	-
W	1.3402(0.0954)	46.0803(3.3754)	-	-

**Table 7:** Selection criteria statistics for dataset 2.

Distribution	-2log	AIC	AD	CvM	K-S	p(AD)	p(CvM)	p(K-S)
GMO-EE	**1079.69**	1087.69	**0.2521**	**0.0338**	**0.0468**	**0.9692**	**0.9628**	**0.9615**
EWP	1082.47	1090.47	0.5490	0.0875	0.0748	0.6971	0.6508	0.5354
EOWEx	1082.68	1088.68	0.4902	0.0694	0.0691	0.7564	0.7557	0.6372
MOEBXII	1090.12	1096.12	0.6516	0.0824	0.0677	0.0600	0.6786	0.6616
GBE-II	1082.79	1088.79	0.6850	0.1173	0.0846	0.5712	0.5081	0.3785
EW	1082.41	1088.41	0.5480	0.0878	0.0750	0.6980	0.6496	0.5310
EE	1082.79	**1086.79**	0.6844	0.1170	0.3788	0.5716	0.5081	0.3787
W	1085.22	1089.22	0.9028	0.1546	0.0900	0.4123	0.3764	0.3050

### 5.3. Dataset 3

**Fig 5:**
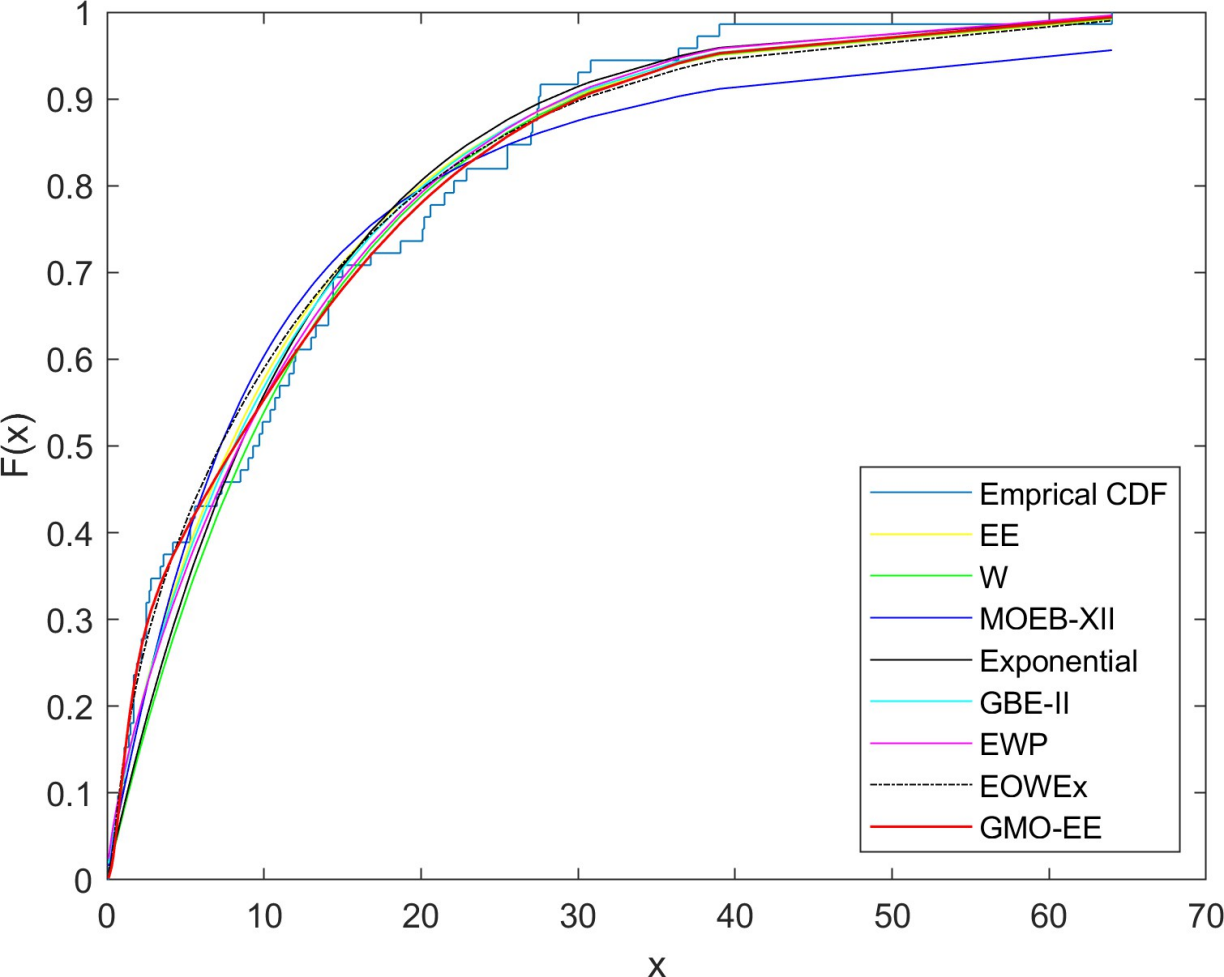
Fitted cdf plots for dataset 3.

The third real data set contains the exceedances of flood peaks (in m^3^/s) of the Wheaton River near Carcross in Yukon Territory, Canada. These data were used in previous studies [45, 46]. The dataset consists of the following values: 1.7, 2.2, 14.4, 1.1, 0.4, 20.6, 5.3, 0.7, 1.9, 13.0, 12.0, 9.3, 1.4, 18.7, 8.5, 25.5, 11.6, 14.1, 22.1, 1.1, 2.5, 14.4, 1.7, 37.6, 0.6, 2.2, 39.0, 0.3, 15.0, 11.0, 7.3, 22.9, 1.7, 0.1, 1.1, 0.6, 9.0, 1.7, 7.0, 20.1, 0.4, 2.8, 14.1, 9.9, 10.4, 10.7, 30.0, 3.6, 5.6, 30.8, 13.3, 4.2, 25.5, 3.4, 11.9, 21.5, 27.6, 36.4, 2.7, 64.0, 1.5, 2.5, 27.4, 1.0, 27.1, 20.2, 16.8, 5.3, 9.7, 27.5, 2.5, and 27.0. We analyze this dataset to compare the GMO-EE with the EE [24], W, MOEBXII [41], GBE-II [42], EOWEx [18], EWP [12], and exponential distributions. The analysis results are given in Tables 8 and 9, and cdf plots of the fitted distributions are shown in Fig 5. As seen from Table 9, the best fitted model is the GMO-EE for all selection criteria.

**Table 8:** Parameter estimates (standard errors) for dataset 3.

	$\hat{\alpha }$	$\hat{\lambda }$	$\hat{\theta }$	$\hat{\beta }$
GMO-EE	0.0112(0.0095)	0.3677(0.0732)	0.0851(0.1238)	2.0726(0.3967)
EWP	32.4750(32.2928)	0.0533(0.0226)	1.3215(0.5396)	0.0176(0.0193)
EOWEx	1.8790(0.5583)	0.4776(0.2163)	12.9329(8.4529)	-
MOEBXII	482.0154(3063.9710)	0.3355(0.4252)	5.7285(8.8558)	-
GBE-II	0.7829(0.1500)	0.0923(0.0217)	0.4838(0.4012)	-
EE	0.0724(0.0117)	0.8284(0.1231)	-	-
W	0.9012(0.0856)	11.6322(1.6017)	-	-
Exponential	0.0819(0.0097)	-	-	-

**Table 9:** Selection criteria statistics for dataset 3.

Distribution	-2log	AIC	AD	CvM	K-S	p(AD)	p(CvM)	p(K-S)
GMO-EE	**494.58**	**502.58**	**0.2486**	**0.0323**	**0.0529**	**0.9710**	**0.9686**	**0.9876**
EWP	502.56	510.56	0.6627	0.1080	0.1079	0.5903	0.5481	0.3718
EOWEx	498.22	504.22	0.4913	0.0891	0.0865	0.7551	0.6424	0.6544
MOEBXII	513.00	519.00	1.2564	0.2045	0.1048	0.2469	0.2593	0.4082
GBE-II	502.26	508.26	0.6976	0.1189	0.1044	0.5603	0.5008	0.4127
EE	506.59	506.59	0.7447	0.1300	0.1017	0.5221	0.4576	0.4462
W	502.99	506.99	0.8445	0.1489	0.1052	0.4496	0.3966	0.4029
Exponential	504.26	506.26	1.4587	0.2306	0.1422	0.1867	0.2153	0.1087

### 5.4. Dataset 4

**Fig 6:**
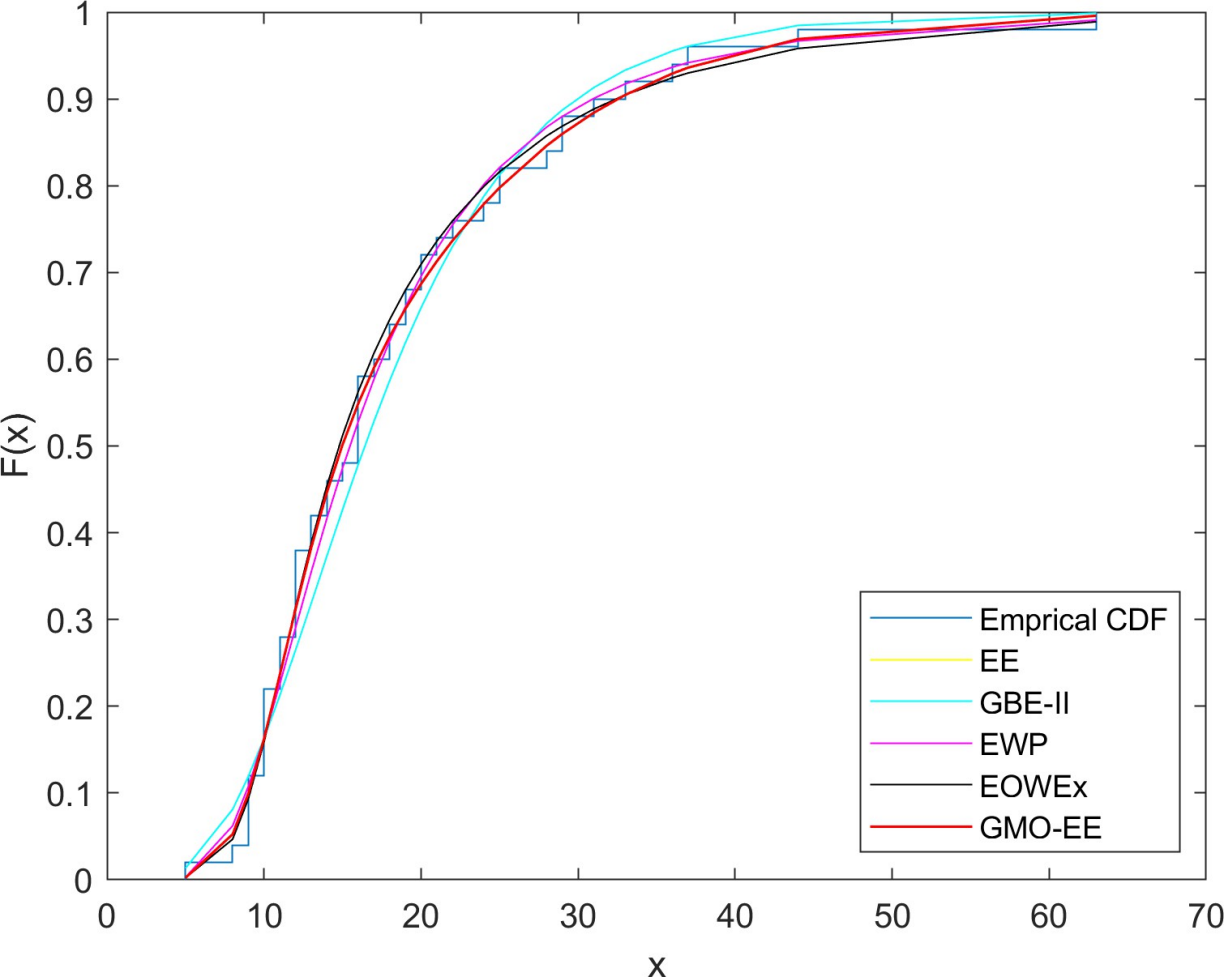
Fitted cdf plots for dataset 4.

The fourth real dataset includes recovery times of survivors as measured from the first positive COVID-19 PCR test to the first negative test for 50 males over 60 years old. This dataset was obtained from anonymized data published by the Israeli Ministry of Health and it was analyzed in a previous work [47]. The dataset contains the following values: 16, 16, 16, 14, 36, 9, 10, 11, 8, 9, 12, 10, 22, 5, 11, 17, 20, 12, 29, 12, 15, 25, 25, 24, 18, 13, 44, 14, 20, 19, 11, 10, 18, 21, 31, 9, 29, 12, 10, 10, 13, 12, 19, 33, 37, 16, 63, 9, 28, and 16. We analyze this dataset to compare the GMO-EE with the W, EE [24], MOEBXII [41], GBE-II [42], EOWEx [18], EWP [12], and exponential distributions. Analysis results are given in Tables 10 and 11, and cdf plots of the fitted distributions are shown in Fig 6.

**Table 10:** Parameter estimates (standard errors) for dataset 4.

	$\hat{\alpha }$	$\hat{\lambda }$	$\hat{\theta }$	$\hat{\beta }$
GMO-EE	0.0551(0.0547)	0.6796(0.1964)	0.1079(0.0280)	9.8323(3.6896)
EWP	16.5628(51.4257)	37.6783(213.0973)	0.3198(0.1848)	90.9496(665.3290)
EOWEx	5.6545(2.0157)	0.0585(0.0065)	4.9490(2.4912)	-
MOEBXII	1010.5069(506.7894)	4.5381(18.0703)	0.5637(2.2449)	-
GBE-II	6.1158(1.7533)	0.1360(0.0302)	0.00002(0.3456)	-
EE	0.1360(0.0189)	6.1153(1.7532)	-	-
W	1.8779(0.1860)	20.8508(1.6693)	-	-
Exponential	0.0544(0.0077)	-	-	-

**Table 11:** Selection criteria statistics for dataset 4.

Distribution	-2log	AIC	AD	CvM	K-S	p(AD)	p(CvM)	p(K-S)
GMO-EE	**346.78**	354.78	**0.2113**	**0.0276**	**0.0690**	**0.9870**	**0.9843**	**0.9712**
EWP	348.21	356.21	0.2972	0.0417	0.0895	0.9400	0.9261	0.8183
EOWEx	346.79	**352.79**	0.2385	0.0350	0.0833	0.9761	0.9586	0.8787
MOEBXII	357.80	363.80	1.3315	0.1816	0.1739	0.2223	0.3069	0.0971
GBE-II	352.10	358.10	0.6886	0.1115	0.1155	0.5677	0.5329	0.5176
EE	352.10	356.10	0.6886	0.1115	0.1155	0.5677	0.5329	0.5176
W	363.40	367.40	1.4451	0.2290	0.1465	0.1902	0.2179	0.2333
Exponential	391.13	393.13	6.3070	1.2118	0.3472	0.0007	0.0007	0.0000

As seen from Table 11, the best fitted model is the GMO-EE according to the -2log, AD, CvM, K-S, p(AD), p(CvM), and p(K-S) criteria, excluding the AIC value of the EOWEx. Satisfactory and comparable model fits are also provided by the three-parameter EOWEx and four-parameter EWP.

## 6. Conclusion

In this study, we have introduced the GMO-EE distribution with (*α*, *λ*, *θ*, *β*) parameters as a sub-model of the GMO distribution family. We have obtained some statistical properties of the new model, such as the moment-generating function, moments, incomplete moments, and Lorenz and Bonferroni curves. Since the GMO-EE distribution has hazard ratio functions with the shape of an upside-down bathtub, bathtub-shaped, increasing, decreasing, constant, and increasing-decreasing-increasing as depicted in Fig 2 for different parameter values, it can be regarded as a flexible distribution for modeling. Moreover, we have provided five different estimation methods for the unknown parameters of the GMO-EE distribution and conducted a Monte Carlo simulation study to evaluate the performances of the estimators. According to the simulation results, the mean biases and MSEs decrease progressively as sample sizes increase.

Four real datasets were fitted to the GMO-EE and some competing distributions to compare them in terms of model fits. The GMO-EE was found to be the best fitted model according to -2log, AD with its p(AD), CvM with its p(CvM), and K-S with its p(K-S) criteria among the competing distributions. When the data on COVID-19 recovery time (dataset 4) were fitted to the GMO-EE distribution, the mean recovery time of male patients aged >60 years was estimated to be 18.35 days. Based on the same dataset, Tanış [47] estimated the mean recovery time to be about 21 days, while Voinsky et al. [48] reported an average recovery time of approximately 15 days for another sample of male COVID-19 patients over the age of 60 years (n = 582) and Barman et al. [49] obtained the 95% confidence interval for average recovery time of 16 to 34 days based on another sample of COVID-19 patients (n = 221). The probability of recovery within 2 weeks was calculated as 44.62% based on the GMO-EE distribution, while Tanış [47] found it to be 45.25%. The results obtained from the GMO-EE distribution are thus supported by the findings of previous studies. From the satisfactory results of these real data applications, the applicability of the GMO-EE model in real life is clear.

In light of our results, we anticipate that the proposed model can be used to fit data obtained from a broad range of fields including survival analysis, meteorology, economics, biology, hydrology, and other applications in life sciences and engineering. Although there are more parsimonious models in the literature, the GMO-EE may still be used effectively thanks to its upside-down bathtub and bathtub-shaped HRFs for modeling biological, clinical, and mortality data in particular. Moreover, the proposed model can be considered as an alternative to the extensions of exponential and Weibull distributions. Further studies based on the GMO-EE distribution could address topics such as parameter estimation of censored data and lifetime regression.
